# Viral metagenomics in mosquitoes as potential vectors of arboviruses in the Colombian Caribbean: characterisation of a “core” regional RNA virome

**DOI:** 10.1590/0074-02760250131

**Published:** 2026-01-12

**Authors:** Richard Hoyos-López, Daniel Echeverri-De la Hoz, Caty Martínez-Bravo, Bertha Gastelbondo-Pastrana, Maira Alemán-Santos, Evelin Garay, Yesica López, Héctor Contreras, Ketty Galeano, German Arrieta, Salim Mattar

**Affiliations:** 1 Universidad de Córdoba, Instituto de Investigaciones Biológicas del Trópico, Córdoba, Colombia; 2 Universidad de Santander, Facultad de Ciencias Médicas y de la Salud, Grupo de Investigación en Ciencias, Valledupar, Cesar, Colombia

**Keywords:** viral metagenomics, next generation sequencing, mosquitoes, arboviruses, virus diversity

## Abstract

**BACKGROUND:**

Mosquitoes are critical vectors in tropical regions where arboviruses like dengue and Zika are prevalent. This study focuses on characterising the RNA virome of mosquitoes in the Colombian Caribbean, emphasising the core regional virome and its role in the dynamics of arboviruses.

**OBJECTIVES:**

The objective was to identify and analyse the core RNA virome of mosquitoes across different genera and seasons in the Colombian Caribbean to understand its composition and potential influence on arbovirus transmission dynamics.

**METHODS:**

In 2023, 4,074 mosquitoes from the genera *Mansonia*, *Coquillettidia*, and *Anopheles* were collected across Córdoba, Sucre, Bolívar, and Magdalena during rainy and dry seasons. Specimens were pooled in groups of 50, subjected to RNA extraction, and sequenced on the MGI-G50™ platform. Bioinformatic analyses utilised the DIAMOND-MEGANizer pipeline and R packages (phyloseq, vegan, ggplot2) to identify viral communities.

**FINDINGS:**

The analysis identified 22 viral families and 24 unclassified RNA viruses. The core regional virome, consistently present across species and seasons, was dominated by insect-specific viruses (ISVs) such as *Aedes aegypti* to virus 1 and 2, Astopletus, and Cumbaru, alongside Picornaviridae (30% of reads), Rhabdoviridae (20%), Orthomyxoviridae, and Bunyavirales. *Mansonia titillans* (38 species) and *Coquillettidia nigricans* (21 species) exhibited the highest viral richness. No significant arboviruses were detected, highlighting ISV dominance. Virome composition varied seasonally, with greater diversity in the rainy season due to increased breeding site availability and temperature.

**MAIN CONCLUSIONS:**

The stability of the core virome suggests it modulates vector competence, potentially reducing arbovirus transmission. These findings advocate the use of metagenomics for enhanced vector surveillance and biological control strategies in neotropical ecosystems.

Colombia is a country with diverse ecosystems that support endemic zones for a wide range of mosquito species and hematophagous habits, allowing the spread of diseases, including malaria and arboviruses such as dengue (DENV), yellow fever (YFV), Venezuelan equine encephalitis (VEEV), and other viruses with historically low transmission rates.^1^ Many of these arboviruses, which are transmitted by mosquitoes, pose significant public health challenges, particularly in tropical and subtropical areas.^2^ In Colombia, the primary families of medically significant arboviruses include Flaviviridae (*e.g.*, *Flavivirus*), *Togaviridae* (*e.g.*, *Alphavirus*), and *Bunyaviridae* (*e.g.*, *Orthobunyavirus* and *Phlebovirus*).^3^ ^,^ ^4^ Arboviruses, such as the West Nile virus (WNV), Saint Louis encephalitis virus (SLEV), Zika virus (ZIKV), and Chikungunya virus (CHIKV), are frequently associated with human diseases in South America. Suspected vectors of arboviruses in Colombia include genera such as *Aedes*, *Anopheles*, *Culex*, and *Haemagogus*, as well as members of the tribe *Mansoniini* and *Sabethini*, which have demonstrated potential roles in arbovirus transmission^3^ ^,^ ^4^ ^,^ ^5^ and are associated with preserved ecosystems and rural areas.^6^ ^,^ ^7^ ^,^ ^8^ ^,^ ^9^ ^,^ ^10^ However, due to ecological changes, such as habitat fragmentation,^10^ the diversity of bats, rodents, primates, avians, marsupials,^11^ ^,^ ^12^ ^,^ ^13^ ^,^ ^14^, and urban expansion,^13^ there is an increased frequency of human-vector contact, increasing the likelihood of outbreaks of emerging and re-emerging arboviruses.^11^ ^,^ ^12^ ^,^ ^13^ ^,^ ^14^

Recent advances in next-generation sequencing (NGS) and metagenomic approaches have transformed the detection of viruses within mosquito populations,^15^ the discovery of new viruses,^16^ the monitoring of emerging and re-emerging arboviruses,^17^ and the enabling of comprehensive virome profiling of individual vectors.^18^ Advances in viral metagenomics applied to mosquitoes have led to the discovery of a vast number of viruses, expanding our understanding of their diversity, classification, and the various environmental conditions in which these viral agents can persist in these vectors.^19^ In addition to arboviruses, a representative group within the mosquito virome, known as insect-specific viruses (ISVs), has been identified.^20^ ^,^ ^21^ These viruses naturally infect arthropods but cannot replicate in vertebrate cells or infect humans, suggesting a long-term symbiotic relationship with their hosts.^22^ In fact, it has been proposed that ISVs play a significant role in modulating vector competence and could be a key component in the design of new strategies for arbovirus control.^23^ Batson et al. used metagenomic sequencing to detect 24 known viral species and 46 novel species in mosquito populations, revealing a diverse viral community that included members of *Reoviridae*, *Picornavirales*, and *Flaviviridae*.^24^ Other studies have applied NGS techniques to analyse viral diversity and identify numerous viral families within mosquito populations across different regions, including *Flaviviridae*, *Togaviridae*, *Phasmaviridae*, and *Phenuiviridae*.^25^ These findings underscore the potential of metagenomic tools for the early detection of both known and novel arboviruses with zoonotic potential.^19^ ^,^ ^21^

Although metagenomic surveillance of arboviruses has been widely used in several countries, its application in Colombia remains relatively limited.^9^ ^,^ ^26^ Although specific viruses have been genomically monitored in some Colombian mosquito populations,^14^ ^,^ ^27^ comprehensive virome studies targeting potentially zoonotic viruses circulating in Colombia, particularly in the Caribbean, are sparse.^9^ ^,^ ^26^ The Colombian Caribbean is a strategic region for arbovirus research due to its high diversity of mosquito vectors - including *Aedes*, *Anopheles*, *Culex*, *Mansonia*, and *Coquillettidia* - and the heterogeneity of its habitats, such as wetlands, mangroves, gallery forests, agricultural areas, and peri-urban environments.^3^ ^,^ ^6^ ^,^ ^8^ ^,^ ^10^ ^,^ ^12^ ^,^ ^14^ These ecological conditions favour the persistence and transmission of emerging and re-emerging viruses, and facilitate interactions between mosquitoes and a wide range of potential reservoirs, including migratory birds - implicated in the spread of WNV and SLEV across the continent -, bats, rodents, and other wild mammals.^1^ ^,^ ^3^ ^,^ ^5^ ^,^ ^8^ ^,^ ^10^ ^-^ ^14^ In this sense, the departments of Cordoba, Sucre, Bolivar, and Magdalena, combine factors that make them a priority for entomovirological surveillance: (i) a history of circulation of arboviruses other than DENV, ZIKV, and CHIKV, including serological and molecular reports of WNV, SLEV, and YFV; (ii) the presence of aquatic ecosystems and flood-prone areas that sustain large mosquito populations; (iii) intense interaction between natural areas and agricultural or livestock landscapes, increasing human-vector contact; and (iv) their location within an ecological and migratory connectivity corridor that may facilitate the introduction and spread of pathogens.^28^ ^,^ ^29^

Worldwide studies have shown that metagenomics is a valuable tool for viral surveillance and biodiversity assessment, demonstrating advantages over traditional virus detection methods in identifying a broader range of viruses.^15^ ^-^ ^19^ ^,^ ^21^ ^,^ ^24^ In tropical settings such as Colombia, metagenomics holds great promise for monitoring the dynamics, diversity, and ecology of mosquito-borne viruses. Conducting virome studies using this metagenomic approach in this Colombian region could provide valuable data on viral diversity and possible emerging and re-emerging viral pathogens, which is fundamental to viral surveillance strategies and could contribute to early detection efforts and strengthen preventive public health measures to mitigate the risk of arbovirus outbreaks in the country.

This study aimed to characterise the RNA virome of mosquito vectors of arboviruses, such as *Mansonia titillans*, *Coquillettidia nigricans*, *Anopheles albimanus*, *Anopheles darlingi*, *Culex nigripalpus*, and *Culex quinquefasciatus*, which are highly abundant in ecosystems related to the Colombian Caribbean, through metagenomic sequencing.

## MATERIALS AND METHODS

*Sampling sites* - The sites included Moñitos (Córdoba) (9º14’51.2”N 76º07’31.2”W), Colosó (Sucre) (9º29’30.7”N 75º21’07.6”W), Talaigua Nuevo (Bolívar) (9º18’08.4”N 74º33’59.0”W), and Santa Ana (Magdalena) (9º19’15.3”N 74º34’33.2”W). These sites were selected based on a combination of ecological, epidemiological, and land-use criteria. Ecologically, they encompass a variety of habitats - including wetlands, mangroves, gallery forests, agricultural lands, and peri-urban areas - that sustain high mosquito diversity (*Aedes*, *Anophele*s, *Culex*, *Mansonia*, *Coquillettidia*) and provide breeding conditions favourable for arbovirus circulation. Epidemiologically, these departments have documented the presence of arboviruses beyond DENV, ZIKV, and CHIKV, including serological and molecular evidence of WNV, SLEV, and yellow fever virus (YFV), reflecting their potential as hotspots for emerging and re-emerging vector-borne pathogens.^3^ ^,^ ^5^ ^,^ ^10^

The selected sites are also situated along important migratory bird routes and contain diverse vertebrate fauna such as bats, rodents, and other mammals, which may act as reservoirs or amplifying hosts for arboviruses.^11^ ^,^ ^12^ ^,^ ^13^ Anthropogenic factors, including wetland fragmentation, rice and monoculture expansion, and cattle ranching, increase human-vector contact and may enhance the likelihood of viral spillover.^3^ ^,^ ^5^ Additionally, the departments’ land-use patterns, agricultural activity, and presence of aquatic ecosystems prone to seasonal flooding create ideal conditions for sustaining large mosquito populations.^14^

Vector sampling was conducted between February and October 2023, with two main field campaigns in each department, timed to coincide with the transitional periods before and after the rainy and dry seasons characteristic of the Caribbean climate. The dry-to-rainy season transition was sampled between February and April 2023, while the rainy-to-dry season transition was sampled between September and October 2023.

In each location, the sampling effort included eight CDC light traps operated for 12 h during the night (18:00-06:00), complemented by active searches carried out by teams of three-four trained personnel using mouth aspirators at potential resting sites among vegetation during early morning (06:00-10:00) and late afternoon (15:00-18:00) hours. Additionally, a Shannon trap was operated from 18:00 to 21:00, during which the same three-four trained personnel performed active mosquito collections using mouth aspirators to capture the specimens attracted to the light.

*Preservation of field-collected mosquito specimens* - Insect capture was performed using CDC light traps, manual capture was carried out with entomological nets and suction pumps from 07:00 to 17:00 for diurnal species, and Shannon traps were placed between 18:00 and 22:00 with light-emitting diode (LED) lights. The captured specimens were separated from non-Culicidae insects, stored in cryovials to the lowest possible taxonomic category, and transported in liquid nitrogen to the Tropical Biological Research Institute (IIBT) at the University of Córdoba, Colombia. Upon arrival, the samples were organised into cryoboxes and stored at -60ºC to -80ºC until taxonomic identification.

*Taxonomic identification of mosquitoes* - Identification was carried out using specialised taxonomic keys for Neotropical Culicidae^30^ ^-^ ^39^ in a climate-controlled room (16ºC) under stereomicroscopes, with specimens placed on chilled trays to preserve their morphological integrity. Mosquitoes were identified to the lowest possible taxonomic level, representative specimens of each morphospecies were set aside and re-examined to verify key diagnostic characters, ensuring consistency and accuracy across all samples. For morphologically challenging groups (*e.g.*, *Culex* subgenus *Melanoconion*), identification included the evaluation of multiple diagnostic traits, and when male specimens were available, genitalia were dissected and analysed to confirm species-level assignments. Although morphological identification served as the primary method for host confirmation, metagenomic sequencing data were also screened for mosquito-specific genetic markers. A subset of quality-filtered reads from each pool was aligned (data no shown) against reference mitochondrial cytochrome oxidase I (COI) sequences and whole-genome scaffolds of relevant mosquito species available in the NCBI database. This cross-validation step was particularly useful for species complexes and ecologically overlapping taxa.

Mosquitoes were pooled by species, considering factors such as sampling date, season (rainy or dry), location, and trap type. Each sample was further separated by daytime or night-time capture, habitat characteristics (riverbanks, residential and peri-domestic areas, and vegetation presence or absence), and trap type, allowing detailed data on the location of mosquito populations and potential viral vectors. The identified females were separated into 1.5 mL tubes per municipality and species to form pools of 50 individuals, which were stored at -80ºC until nucleic acid extraction.

*RNA Extraction, library preparation, and sequencing* - Mosquito pools of 50 individuals were used to ensure sufficient viral RNA detection and high-quality metagenomic libraries. Pooling enhances sensitivity for low-abundance viruses, which may be below detection limits in individual mosquitoes, and provides adequate RNA yield for robust sequencing. The mosquito cells were triturated in cold mort µL of Dulbecco’s minimum essential medium supplemented with 10% foetal bovine serum (FBS) and 1% penicillin and clarified by centrifugation at 13000 rpm for 30 min. From the resulting supernatant (400 µL), was filtered through a 0.22 μm membrane, and nucleic acids were extracted using the MagMAX™ Viral/Pathogen Nucleic Acid Isolation Kit (Thermo Scientific, Waltham, Massachusetts, United States), following the manufacturer’s instructions. For sequencing, the RNA concentration and integrity number (RIN) were measured fluorometrically using a QubitTM Broad Range (BR) RNA Quantification Kit and an RNA IQÔ Assay Kit (Thermo Fisher Scientific). An input of 250-500 ng total RNA was used for library preparation. Samples were processed using the MGIEasyÔ Fast RNA library preparation set and high-throughput DNA nanobead (DNB) technology. RNA was fragmented into approximately 250 nucleotides using the FCL 150 paired-end (PE) platform. The first and second DNA strands were synthesised using random hexamer primers. The fragments were subjected to end-catalytic repair (ERAT), molecular barcode ligation, and product amplification using polymerase chain reaction (PCR). The library concentration was determined using the QubitTM dsDNA Quantification Assay Kit, and the fragment size was determined using a Fragment AnalyzerTM (Agilent Technologies). Pools were obtained for DNA circularisation and DNB synthesis (> 11 ng/μL), based on the concentration and size of the fragments. Next-generation sequencing was performed using DNB on an MGI-G50TM equipment (Shenzhen, China).

*Bioinformatic analysis* - This viral metagenomic analysis was conducted using the DIAMOND-MEGANizer pipeline,^40^ ^,^ ^41^ providing an efficient approach for taxonomic and functional classification of large metagenomic datasets by combining DIAMOND’s rapid sequence alignment capabilities with MEGANizer’s precise taxonomic binning. Initially, raw reads were preprocessed by quality filtering and trimming using Fastp to eliminate adapters and low-quality bases (minimum quality score of 20 and a read length of 50 bp). Subsequently, Bowtie2 was used to remove host genome contamination, allowing the focus to remain on the viral and microbial sequences. *De novo* assembly, with a minimum length of 300 nucleotides, was performed using the MEGAHIT software.^41^ Subsequently, the filtered reads were aligned to the NCBI non-redundant (nr) protein database using DIAMOND in the BLASTx mode. DIAMOND was configured with an E-value threshold of 1e-5, limiting target sequences to one to focus on the best hit and using a sensitive mode for optimal capture of diverse viral sequences. The alignment output was saved in DAA format (DIAMOND Alignment Archive (DAA) format, which is ideal for downstream taxonomic analysis using MEGANizer.

The MEGANizer module of MEGAN6 was used to taxonomically classify DIAMOND alignment results. MEGANizer employs NCBI taxonomy and mapping files to assign reads to taxonomic groups and applies the Lowest Common Ancestor (LCA) algorithm to ensure robust assignments. Customised filtering thresholds, such as a minimum bit score of 50 and requiring at least 50% read coverage, enhanced the accuracy of the assignment. These taxonomic classifications provided an in-depth profile of the viral and microbial community composition, identifying the prevalent viral families and genera.

Diversity and abundance metrics were calculated to analyse the viral community structures across the samples. The R programming language with packages such as phyloseq, vegan, and ggplot2 was used to visualise the taxonomic and functional profiles.^42^ To ensure robust and accurate diversity estimates, raw sequence data were quality-filtered and normalised prior to analysis. Alpha diversity metrics were calculated for within-sample viral richness and evenness. Beta diversity was assessed using Bray-Curtis dissimilarity indices to measure differences in viral community composition between samples. The Bray-Curtis dissimilarity index was chosen for beta diversity to account for both presence/absence and relative abundance of viruses, critical for viromes with high abundance variation and sparsity.

Alpha and beta diversity metrics allowed for comparisons of viral diversity across samples, localities, mosquito species, and sampling periods. Hierarchical clustering and heatmaps further highlighted the similarities and differences in viral assemblages among the samples. Additionally, taxon abundance tables were generated to quantify the presence and distribution of viral families, allowing for a comparative analysis between mosquito species and environmental conditions. The final visualisations displayed taxonomic summaries, functional analyses, and abundance heatmaps, facilitating the interpretation of viral dynamics in environmental samples.^43^

## RESULTS

A total of 4,074 mosquitoes were collected, and pools were organised into 33 samples corresponding to the mosquito species previously selected based on abundance (Table). 1,729 were captured during the dry season and 2,345 during the rainy season. The most abundant species identified across the four locations in the Colombian Caribbean included *Ma. titillans*, *Cq. nigricans*, and *An. albimanus*, other relevant species were *Anopheles darlingi*, *Cx. nigripalpus*, and *Cx. quinquefasciatus*.

**TABLE:** Samples sequenced by mosquito species and summary quality data of metagenomic analysis results

Pool (Sample)	Mosquito species	Number of raw reads	Classified reads		Unclassified reads	reads with low quality:		Viral contings	mean length before filtering	mean length after filtering
			Number	%	Number	Number	%	Number		
Pool 1	*Mansonia titillans* ^*^	63.545616 M	63.520688 M	99.960771%	24.928	12.108000 K	0.019054%	25	150bp	147bp
Pool 4	*Ma. titillans*	42.891598 M	42.868378 M	99.945864%	23.220	6.258000 K	0.014590%	135	150 bp	146 bp
Pool 5	*Ma. titillans*	39.183604 M	39.161586 M	99.943808%	22.018	7.540000 K	0.019243%	191	150 bp	146 bp
Pool 6	*Coquillettidia nigricans*	32.510398 M	32.485280 M	99.922739%	25.118	12.782000 K	0.039317%	190	150 bp	146 bp
Pool 7	*Ma. titillans*	34.086056 M	34.068206 M	99.947633%	17.850	5.004000 K	0.014680%	159	150 bp	145 bp
Pool 8	*Ma. titillans*	47.286180 M	47.260626 M	99.945959%	25.554	7.874000 K	0.016652%	227	150 bp	147 bp
Pool 9	*Ma. titillans*	39.441954 M	39.420532 M	99.945687%	21.422	9.008000 K	0.024248%	370		
Pool 10	*Ma. titillans*	39.441954 M	39.420532 M	99.945687%	21.422	6.578000 K	0.016678%	196		
Pool 11	*Ma. titillans*	14.408840 M	14.356840 M	99.639110%	52.000	50.816000 K	0.352672%	102	150 bp	149 bp
Pool 12	*Ma. titillans*	26.695796 M	26.615892 M	99.700687%	79.904	77.848000 K	0.291611%	118	150 bp	147 bp
Pool 13	*Ma. titillans*	31.219236 M	31.139840 M	99.745682%	79.396	77.108000 K	0.246989%	118	150 bp	146 bp
Pool 14	*Ma. titillans*	24.188584 M	24.123974 M	99.732891%	64.610	62.916000 K	0.260106%	82	150 bp	146 bp
Pool 15	*Cq. nigricans*	23.446948 M	23.381252 M	99.719810%	65.696	64.140000 K	0.273554%	75	150 bp	144 bp
Pool 16	*Cq. nigricans*	31.440184 M	31.377876 M	99.801820%	62.308	59.964000 K	0.190724%	25	150 bp	143 bp
Pool 17	*Cq. nigricans*	29.150906 M	28.958474 M	99.339876%	192.432	191.758000 K	0.657811%	382	150 bp	148 bp
Pool 18	*Ma. titilans*	18.289456 M	18.100306 M	98.965798%	189.150	188.612000 K	1.031261%	239	150 bp	146 bp
Pool 19	*Ma. titilans*	13.203390 M	13.079460 M	99.061377%	123.930	123.588000 K	0.936032%	250	150 bp	146 bp
Pool 21	*Anopheles albimanus*	21.596384 M	21.477542 M	99.449713%	118.842	118.276000 K	0.547666%	74	150 bp	148 bp
Pool 22	*An. albimanus*	17.133586 M	17.042842 M	99.470374%	90.744	90.196000 K	0.526428%	53	150 bp	148 bp
Pool 23	*Culex nigripalpus*	22.466642 M	22.392558 M	99.670249%	74.084	73.462000 K	0.326983%	14	150 bp	148 bp
Pool 24	*Cx. nigripalpus*	22.861688 M	22.721658 M	99.387491%	140.030	139.476000 K	0.610086%	50	150 bp	148 bp
Pool 25	*Anopheles darlingi*	35.837204 M	35.631098 M	99.424883%	206.106	205.106000 K	0.572327%	86	150 bp	148 bp
Pool 26	*Anopheles trianulatus*	3.523866 M	3.507178 M	99.526429%	16.688	16.588000 K	0.470733%	26	150 bp	147 bp
Pool 30	*Ma. titilans_*	41.013562 M	40.948962 M	99.842491%	64.600	63.888000 K	0.155773%	374	150 bp	148 bp
Pool 31	*Culex quinquefasciatus*	23.133230 M	23.062480 M	99.694163%	70.750	70.130000 K	0.303157%	44	150 bp	147 bp
Pool 33	*Cx. quinquefasciatus*	44.969288 M	44.886730 M	99.816412%	82.558	81.908000 K	0.182142%	167	150 bp	147 bp
Pool 34	Cx. quinquefasciatus	39.372892 M	39.316710 M	99.857308%	56.182	55.750000 K	0.141595%	39	150 bp	147 bp
Pool 35	*Cx. quinquefasciatus*	44.903198 M	44.824108 M	99.823866%	79.090	78.554000 K	0.174941%	60	150 bp	147 bp
Pool 36	*Cx. nigripalpus*	49.382146 M	49.319874 M	99.873898%	62.272	61.622000 K	0.124786%	78	150 bp	147 bp
Pool 37	*Cx. nigripalpus*	52.652456 M	52.580268 M	99.862897%	72.188	71.618000 K	0.136020%	84	150 bp	146 bp
Pool 38	*Ma. titilans*	45.698564 M	45.659744 M	99.915052%	38.820	38.258000 K	0.083718%	509	150 bp	148 bp

*Species names are presented in full at their first mention. RNA: ribonucleic acid.

**Fig. 1:**
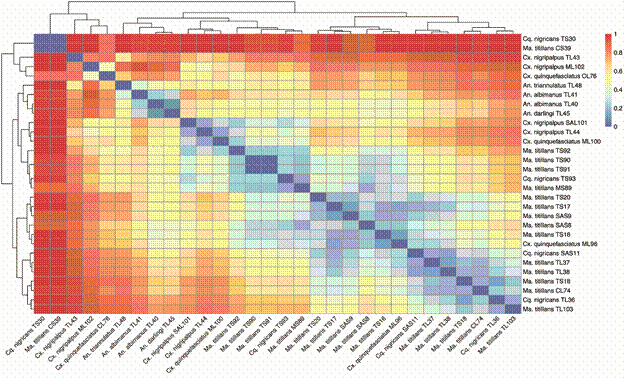
relative abundance of viral families in mosquito species. Bar graph showing viral families in the virome of each mosquito species: *Mansonia titillans*, *Culex quinquefasciatus*, *Culex nigripalpus*, *Coquillettidia nigricans*, *Anopheles triannulatus*, *Anopheles darlingi*, and *Anopheles albimanus*. ISVs: insect-specific viruses. The abundance of viral families was estimated by transforming the number of reads into relative values, which provided an assessment of their presence in each mosquito species.

Bioinformatics analyses revealed contigs associated with 22 viral families and 24 RNA viruses with no current taxonomic classification in the study mosquitoes. ISVs are the most notorious and abundant viruses among the mosquito species studied. In this sense, *Ma. titillans* presented 38 viral species, of which 17 had no taxonomic classification, while 21 viruses belonged to the orders Picornavirales, Bunyavirales, and Mononegavirales, but could not be placed within the families of these orders (Fig. 1). Viruses from the families Picornaviridae and Rhabdoviridae were also identified in the present study. *Cq. nigricans* presented 21 viral species; seven were identified as RNA viruses with no known taxonomic classification, whereas the remaining belonged to the families Orthomyxoviridae, Parvoviridae, Baculoviridae, Nudiviridae, Flaviviridae, Totiviridae, and Metaviridae and the orders Bunyavirales and Mononegavirales.

*Culex quinquefasciatus* and *Cx. nigripalpus* shared a high diversity of unclassified riboviruses and viruses; in fact, *Cx. nigripalpus* is the mosquito species with the most unclassified viruses, with only the Rhabdoviridae family being identified. *Cx. quinquefasciatus* belong to the Metaviridae and Mesoniviridae families. Viral diversity in *An. triannulatus*, *An. darlingi* and *An. albimanus* showed similar viral groups represented by the Artoviridae, Baculoviridae, Metaviridae, and Mesoniviridae families, with Rhabdoviridae viruses detected in *An. manus* (Fig. 1).

**Fig. 2:**
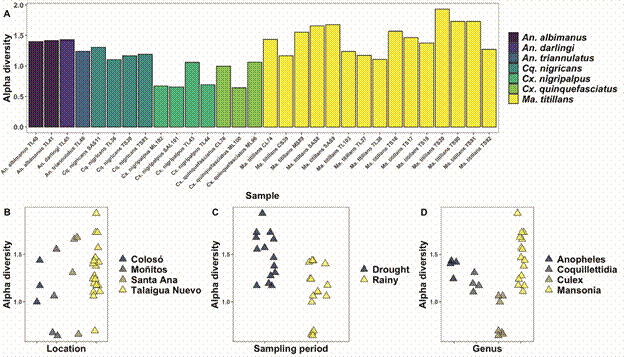
relative abundance of insect-specific virus (ISVs) in mosquito species. Bubble plot showing the relative abundance of ISVs*.* The abundance of the viral species was estimated by transforming the number of reads into relative values, providing an assessment of their presence in each mosquito species.

The ISV most representative was *Aedes aegypti to virus 1* and *Ae. aegypti*, and *virus 2* was identified in all mosquito species studied, followed by Astopletus, *Gordis, Cumbaru*, *Kaiowa*, *Keturi*, *Nefer*, *Nejeret*, and *Wilkie ophio-like virus 1* (Fig. 2). Other viruses with low abundance included *Culex flavivirus*, *Chibugado virus*, and *Atrato Partititi-like virus*.

**Fig. 3:**
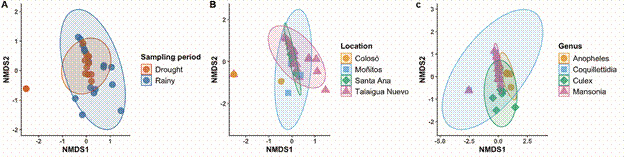
estimation of Alpha diversity using the Shannon-Wiener index (H’) and non-metric multidimensional scaling (NMDS) for the viral diversities found according to mosquito species, sampling locality, and sampling period.

**Fig. 4:**
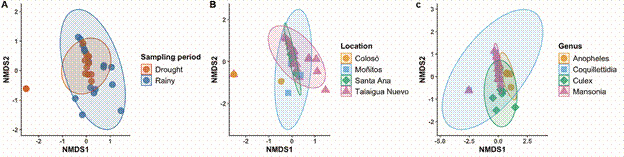
non-metric multidimensional scaling (NMDS) between sampling period, location, and mosquito genus.

**Fig. 5:**
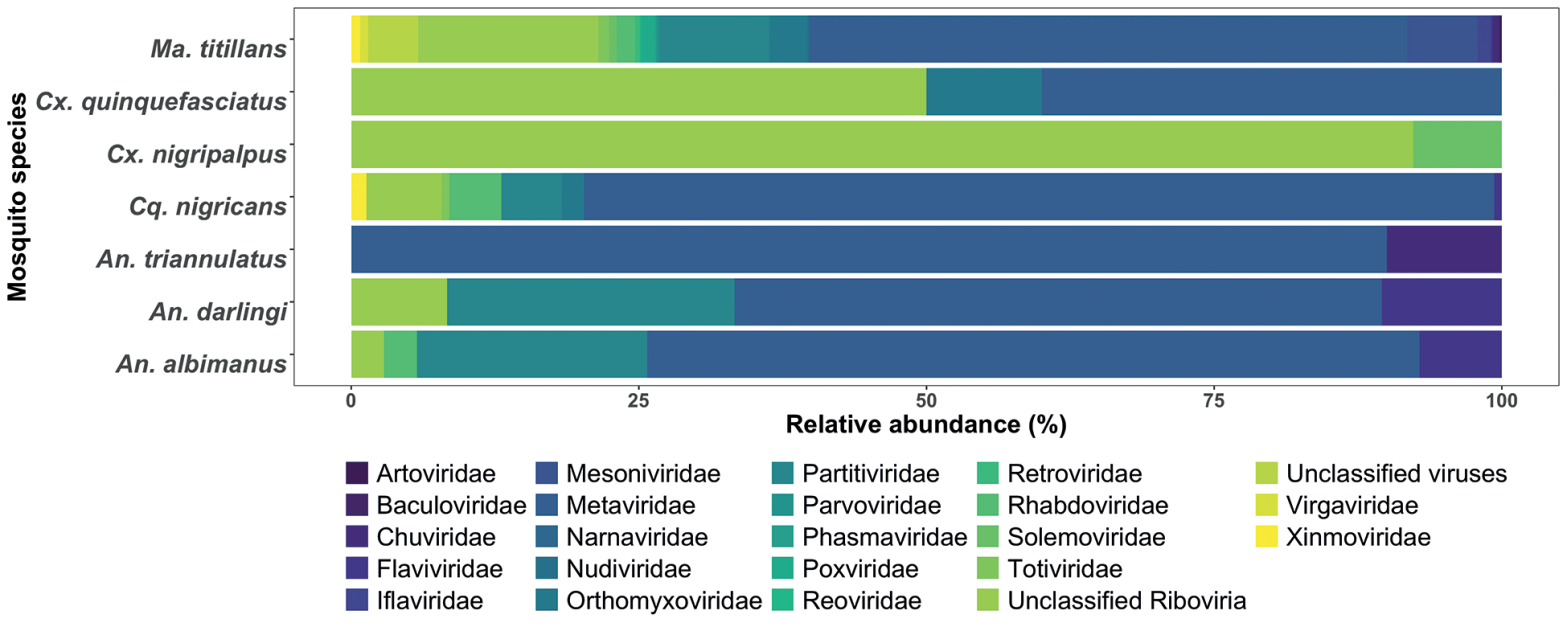
heatmap matrix using a colour scale; the similarity between the viral diversities found in the sequenced samples of each mosquito species.

Regarding viral ecology, when comparing the alpha diversity found in the localities, similarities were observed between Santa Ana/Talaigua Nuevo, Moñitos, and Colosó, along with a segregation in viral diversity results between the dry/rainy seasons, and mosquito genus (Fig. 3). This can be explained by changes in the diversity and abundance of mosquito species collected during both entomological sampling periods (Fig. 4). However, non-metric Multidimensional Scaling (NMDS) of the viral communities found by sampling period and location highlighted a ‘*core*’ of similarity among the viruses present in the different evaluated mosquito species (Fig. 5).

## DISCUSSION

In this study, we analysed the virome of mosquitoes captured in the Colombian Caribbean, where ISVs were the most abundant viruses among all mosquito species analysed. Although these viruses do not pose a direct risk to humans, they can influence mosquito vector competition and the transmission of arboviruses of public health importance. Future research should evaluate the influence of ISVs on vector capacity and their implications for developing mosquito-borne disease control strategies.

Analysis of viral diversity showed that *Ma. titillans* and *Cq. nigricans* harbored the highest viral richness compared with other species. First, the viability of using populations of mosquito species in viral metagenomics was demonstrated, which can provide much more accurate RNA virome profiles, allowing interspecific comparisons. In the present study, similarities were observed between *Ma. titillans* and *Cq. nigricans* in terms of viral abundance and diversity, particularly for Bunyavirales, Metaviridae, and viruses that were not systematically classified. However, for *Cq. nigricans*, differences were observed among the families Flaviviridae, Parvoviridae, Orthomyxoviridae, Baculoviridae and Nudiviridae. These two mosquito species appear to have their own relatively stable “eukaryotic virome,” despite the geographical distances in the study areas, which could have significant implications for their ability to transmit medically important arboviruses to humans.

Exploring the mosquito virome and understanding how its composition influences arbovirus transmission is essential for understanding the emergence of arboviral diseases and the dynamics of their outbreaks. In this study, we performed metagenomic sequencing to characterise the virome of epidemiologically important mosquito species in the Colombian Caribbean region, which has varying ecological conditions that may affect vector-virus interactions and viral diversity. The mosquito species analysed were selected based on their abundance across different localities and seasons, including *Ma. titillans*, *Cq. nigricans*, *An. albimanus*, *An. darlingi*, *Cx. nigripalpus*, and *Cx. quinquefasciatus*.

Our analysis revealed a notable predominance of ISVs in the viromes of all mosquito species studied. These ISVs, which infect insects but not vertebrates, have been identified as potential modulators of arboviral transmission. The presence of ISVs, such as *Ae. aegypti* to virus 1 and *Ae. aegypti* to virus 2 in all mosquito species studied, underscores the importance of these viruses in shaping the dynamics of the mosquito virome. Additionally, ISVs such as *Astopletus*, *Gordis*, *Cumbaru*, *Kaiowa*, *Keturi*, *Nefer*, *Nejeret*, and *Wilkie ophio-like virus 1* been detected with varying prevalence across species. Interestingly, *Cx. flavivirus*, *Chibugado virus*, and *Atrato Partititi-like viruses* were found in lower abundance, suggesting potential geographical or ecological constraints on their distribution. Taxonomic analysis of viral sequences identified a high diversity of viral families associated with mosquitoes, including Picornaviridae, Rhabdoviridae, Orthomyxoviridae, Parvoviridae, Baculoviridae, Nudiviridae, Flaviviridae, Totiviridae, and Metaviridae, as well as unclassified RNA viruses. Notably, *Ma. titillans* harbored the greatest viral diversity, with 38 viral species, including 17 with no taxonomic classification. This highlights the need for further investigation to elucidate the evolutionary relationships and ecological roles of these viruses. Similarly, *Cq. nigricans* exhibits diverse viromes, including members of the orders Bunyavirales and Mononegavirales, which contain arboviruses of medical importance. The high abundance of unclassified Riboviria viruses in *Cx. nigripalpus* suggests a potential reservoir function for novel viruses that requires further investigation.

In previous virome and metagenomic studies on mosquitoes, various viral families were identified in different mosquito species. The specific findings for each species studied in our research and their comparisons with studies in other regions are presented below.

*Mansonia titillans* - In this study, 38 viral species were identified, including members of the families Picornaviridae, Rhabdoviridae, Orthomyxoviridae, Parvoviridae, Baculoviridae, Nudiviridae, Flaviviridae, Totiviridae, and Metaviridae. Previous studies in Brazil have reported the presence of Flaviviridae and Bunyavirales in this species, as well as viruses such as the *Mansonia* flavivirus.^44^ ^,^ ^45^ ^,^ ^46^ ^,^ ^47^

*Coquillettidia nigricans* - Viruses from the orders Bunyavirales and Mononegavirales were detected. In studies from other regions, such as Argentina, Flaviviridae and Rhabdoviridae viruses have been identified, with reports of Coquillettidia-associated viruses.^48^ ^,^ ^49^ ^,^ ^50^ ^,^ ^51^

*Anopheles albimanus* - In this study, viruses from the families Flaviviridae and Rhabdoviridae were identified. Previous studies have documented the presence of Mesoniviridae and Peribunyaviridae in this species in Mexico and Colombia, including Anopheles-associated flaviviruses.^45^ ^,^ ^47^ ^,^ ^52^ ^-^ ^59^

*Anopheles darling* - Primarily flaviviruses and a small number of unclassified viruses were detected. Studies in Brazil and Peru have identified viruses from Totiviridae and Iflaviridae, respectively.^44^ ^,^ ^50^

*Culex nigripalpus* - A high abundance of unclassified Riboviria was observed in this species. In studies from North America and Brazil, Rhabdoviridae, Flaviviridae, and Peribunyaviridae have been reported, including *Cx. nigripalpus* nucleopolyhedrovirus.^48^ ^,^ ^54^ ^,^ ^58^

*Culex quinquefasciatus* - Viruses from Flaviviridae and Rhabdoviridae were detected in this study. Studies from Asia and Africa have recorded Totiviridae, Iflaviridae, and Baculoviridae in this species, with the presence of Culex-associated flaviviruses and Culex-borne rhabdoviruses.^46^ ^,^ ^53^ ^,^ ^56^

The observed differences in viral diversity across mosquito species and localities may be driven by environmental factors such as temperature, humidity, and breeding site availability [Supplementary data (Table)]. Our findings indicate similarities in alpha diversity between the mosquito viromes from Santa Ana/Talaigua Nuevo, Moñitos, and Coloso, whereas a clear segregation was observed between the viromes of mosquitoes collected during the dry and rainy seasons. Alpha diversity analysis indicated significant variability between localities and seasons, highlighting the influence of environmental factors on the structure of the virome. In addition, the segregation observed in the beta diversity analysis (NMDS) suggests that viral composition varies between species and seasons, with an increase in viral richness in the rainy season. This seasonal effect aligns with previous studies, indicating that fluctuations in mosquito population dynamics influence the virome composition. Additionally, NMDS analysis revealed a ‘core’ of similarity among viral communities across different mosquito species, supporting the idea of shared viral reservoirs and horizontal transmission of certain ISVs in the studied region. Similarly, the results showed that Santa Ana and Talaigua Nuevo share a homogeneous viral profile, possibly due to ecological similarities and the presence of nearby bodies of water. Colosó presents a differentiated viral community, which could be due to habitat and the availability of alternative hosts.

From an epidemiological perspective, the presence of diverse viral families, particularly *Rhabdoviridae*, *Flaviviridae*, and *Mesoniviridae*, highlights the potential of mosquitoes to serve as vectors for emerging viruses. Given that *Culex* and *Anopheles* mosquitoes are known to transmit arboviruses of medical importance, their high viral diversity warrants continued surveillance to detect potential spillovers. Moreover, the detection of ISVs with a broad geographic distribution suggests that these viruses may play a role in vector competence, possibly influencing mosquito susceptibility to pathogenic arboviruses.

The implications of these findings extend to vector control strategies and biotechnology. The potential of ISVs as biological control agents has gained attention in recent years because they may interfere with the replication of pathogenic viruses within mosquitoes through mechanisms such as competitive exclusion and immune priming in the host. The identification of common ISVs across multiple vector species reinforces the need to explore their ecological roles and interactions with arboviruses in the future. Additionally, the discovery of unclassified viruses in this study highlights the importance of continued metagenomic research to expand our knowledge of mosquito-associated viromes and their potential applications in vector-borne disease management.

*In conclusion* - Our study provides an in-depth characterisation of the mosquito RNA virome in the Colombian Caribbean region, revealing a rich diversity of ISVs and unclassified RNA viruses. The seasonal and geographic variations observed in viral diversity emphasise the need for longitudinal studies to assess virome stability over time. Further investigations into the ecological and evolutionary dynamics of mosquito-associated viruses are crucial for understanding their roles in arbovirus transmission and vector ecology. Our findings provide valuable insights into the virome composition of Neotropical mosquito species and highlight the potential of ISVs as tools for future vector-control strategies. Future research should focus on the functional characterisation of unclassified viruses, particularly those detected in mosquito species that are known viral pathogen vectors, such as species of the genus *Culex*. In addition, the integration of metagenomic data with ecological and epidemiological studies will allow a broader understanding of the transmission dynamics of arboviruses in the region, development of preventive strategies, and strengthening of the capacity to respond to outbreaks of mosquito-borne diseases in the Colombian Caribbean.

## Supplementary Materials

Supplementary material

## Data Availability

The sequences are available in the Sequence Read Archive (SRA) under the accession number PRJNA1251058.

## References

[1] Ramirez RMG, Bohers C, Mousson L, Madec Y, Vazeille M, Piorkowski G (2024). Increased threat of urban arboviral diseases from Aedes aegypti mosquitoes in Colombia. IJID Reg.

[2] Sanchez-Lerma L, Rojas-Gulloso A, Miranda J, Tique V, Patino LH, Rodriguez D (2024). Unexpected arboviruses found in an epidemiological surveillance of acute tropical febrile syndrome in the department of Meta, Eastern Colombia. J Infect Public Health.

[3] Hoyos-López R, Suaza-Vasco J, Rúa-Uribe G, Uribe S, Gallego-Gómez JC (2016). Molecular detection of flaviviruses and alphaviruses in mosquitoes (Diptera Culicidae) from coastal ecosystems in the Colombian Caribbean. Mem Inst Oswaldo Cruz.

[4] Segura NA, Munoz AL, Losada-Barragan M, Torres O, Rodriguez AK, Rangel H (2021). Epidemiological impact of arboviral diseases in Latin American countries, arbovirus-vector interactions and control strategies. Pathog Dis.

[5] Mattar S, Paternina D, Herazo R, Oviedo M, Rivero R Climatic variables and the reemerging Eastern and Venezuelan equine encephalitis in equines, Colombia 2005-2023. SSRN [Preprint].

[6] Parra-Henao G, Suarez L (2012). Mosquitos (Diptera Culicidae) vectores potenciales de arbovirus en la region de Uraba, noroccidente de Colombia. Biomedica.

[7] Martinez D, Hernandez C, Munoz M, Armesto Y, Cuervo A, Ramirez JD (2020). Identification of Aedes (Diptera Culicidae) species and arboviruses circulating in Arauca, Eastern Colombia. Front Ecol Evol.

[8] Laurito M, Hoyos-López R (2018). First record of Culex (Culex) bidens (Diptera Culicidae) in Colombia: taxonomic and epidemiological implications. Acta Trop.

[9] Gomez M, Martinez D, Paez-Triana L, Luna N, De Las Salas JL.Hernandez C (2023). Characterizing viral species in mosquitoes (Culicidae) in the Colombian Orinoco insights from a preliminary metagenomic study. Sci Rep.

[10] Cano-Pérez E, González-Beltran M, Ampuero JS, Gómez-Camargo D, Morrison AC, Astete H (2022). Prevalence of mosquito populations in the Caribbean region of Colombia with important public health implications. Trop Med Infect Dis.

[11] Calderon A, Guzman C, Mattar S, Rodriguez V, Martinez C, Violet L (2019). Dengue virus in bats from Cordoba and Sucre, Colombia. Vector Borne Zoonotic Dis.

[12] Hoyos J, Carrasquilla MC, Leon C, Montgomery JM, Salyer SJ, Komar N (2021). Host selection pattern and flavivirus screening of mosquitoes in a disturbed Colombian rainforest. Sci Rep.

[13] Gonzalez-Astudillo V, Ramirez-Chaves HE, Henning J, Gillespie TR (2016). Current knowledge of studies of pathogens in Colombian mammals. Manter J Parasite Biodivers.

[14] Lopez RH, Soto SU, Gallego-Gomez JC (2015). Evolutionary relationships of West Nile virus detected in mosquitoes from a migratory bird zone of Colombian Caribbean. Virol J.

[15] Pan YF, Zhao H, Gou QY, Shi PB, Tian JH, Feng Y (2024). Metagenomic analysis of individual mosquito viromes reveals the geographical patterns and drivers of viral diversity. Nat Ecol Evol.

[16] He X, Yin Q, Zhou L, Meng L, Hu W, Li F (2021). Metagenomic sequencing reveals viral abundance and diversity in mosquitoes from the Shaanxi-Gansu-Ningxia region, China. PLoS Negl Trop Dis.

[17] Aragao CF, da Silva SP, do Nascimento BLS, da Silva FS, Nunes JP, Pinheiro VCS (2023). Shotgun metagenomic sequencing reveals virome composition of mosquitoes from a transition ecosystem of North-Northeast Brazil. Genes.

[18] Ramirez AL, Colmant AM, Warrilow D, Huang B, Pyke AT, McMahon JL (2020). Metagenomic analysis of the virome of mosquito excreta. mSphere.

[19] Laiton-Donato K, Guzman-Cardozo C, Pelaez-Carvajal D, Ajami NJ, Navas MC, Parra-Henao G (2023). Evolution and emergence of mosquito-borne viruses of medical importance towards a routine metagenomic surveillance approach. J Trop Ecol.

[20] Vasilakis N, Tesh RB (2015). Insect-specific viruses and their potential impact on arbovirus transmission. Curr Opin Virol.

[21] de Almeida JP, Aguiar ER, Armache JN, Olmo RP, Marques JT (2021). The virome of vector mosquitoes. Curr Opin Virol.

[22] de Faria IJ, de Almeida JP, Marques JT (2024). The impact of symbiotic insect-specific viruses on mosquito vector competence for arboviruses. Curr Opin Insect Sci.

[23] Hall RA, Bielefeldt-Ohmann H, McLean BJ, O'Brien CA.Colmant AM.Piyasena TB (2016). Commensal viruses of mosquitoes host restriction, transmission, and interaction with arboviral pathogens. Evol Bioinform.

[24] Batson J, Dudas G, Haas-Stapleton E, Kistler AL, Li LM, Logan P (2021). Single mosquito metatranscriptomics identifies vectors, emerging pathogens and reservoirs in one assay. Elife.

[25] da Silva AG, Bach E, Ellwanger JH, Chies JAB (2024). Tips and tools to obtain and assess mosquito viromes. Arch Microbiol.

[26] Martinez D, Gomez M, Hernandez C, Florez AZ, Munoz M, Ramirez JD (2023). Employing Oxford Nanopore Technologies (ONT) for understanding the ecology and transmission dynamics of flaviviruses in mosquitoes (Diptera Culicidae) from Eastern Colombia. Acta Trop.

[27] Hoyos-López R, Soto SU, Rúa-Uribe G, Gallego-Gómez JC (2015). Molecular identification of Saint Louis encephalitis virus genotype IV in Colombia. Mem Inst Oswaldo Cruz.

[28] Weaver SC (2018). Prediction and prevention of urban arbovirus epidemics a challenge for the global virology community. Antiviral Res.

[29] Campos RK, Rossi SL, Tesh RB, Weaver SC (2023). Zoonotic mosquito-borne arboviruses spillover, spillback, and realistic mitigation strategies. Sci Transl Med.

[30] Lane J (1953). Neotropical Culicidae. USP.

[31] Cova García P, Sutil E, Rausseo JA (1966). Mosquitoes of Venezuela.

[32] Bram RA (1967). Classification of Culex subgenus Culex in the New World (Diptera Culicidae). Proc US Nat Hist Mus.

[33] Arnell JH (1973). Mosquito studies (Diptera Culicidae) XXXII. A revision of the genus Haemagogus. Contrib Am Entomol Inst.

[34] Berlin O, Belkin J (1980). Mosquito studies (Diptera Culicidae) XXXVI. Subgenera Aedinus, Tinolestes and Anoedioporpa of Culex. Contrib Am Entomol Inst.

[35] Clark-Gil S, Darsie RF (1983). The mosquitoes of Guatemala their identification, distribution and bionomics. Mosq Syst.

[36] Forattini O (2002). Culicidologia medica: identificacao, biologia, epidemiologia. II.

[37] Gonzalez R, Carrejo N (2007). Introduccion al estudio taxonomico de Anopheles de Colombia. Univ del Valle.

[38] Pecor JE, Mallampalli VL, Harbach RE, Peyton EL (1992). Catalog and illustrated review of the subgenus Melanoconion of Culex (Diptera Culicidae). Contrib Am Entomol Inst.

[39] Sirivanakarn S (1982). A review of the systematics and proposed scheme of internal classification of the New World subgenus Melanoconion of Culex (Diptera Culicidae). Mosq Syst.

[40] Gautam A, Zeng W, Huson DH, Nagarajan N, Pop M (2023). Metagenomic data analysis.

[41] Bagci C, Patz S, Huson DH (2021). DIAMOND+ MEGAN fast and easy taxonomic and functional analysis of short and long microbiome sequences. Curr Protoc.

[42] Seah BK, Gruber-Vodicka HR gbtools: interactive visualization of metagenome bins in R (2015). Front. Microbiol.

[43] Huson DH, Beier S, Flade I, Gorska A, El-Hadidi M, Mitra S (2016). MEGAN community edition interactive exploration and analysis of large-scale microbiome sequencing data. PLoS Comput Biol.

[44] De Sousa FB, Valencia JA, Ramos AP, Naveca FG, Silva GA, Abrahim CM (2023). Report of natural Mayaro virus infection in Mansonia humeralis (Dyar & Knab, Diptera Culicidae). Parasit Vectors.

[45] Miranda KK, Ribeiro-Junior GF, Sacramento CQ, Mota TM, Souza DM, Lemos PS (2022). Discovery and genome sequencing of a new virus related to members of the family Tymoviridae, isolated from mosquitoes of the genus Mansonia in Brazil. Arch Virol.

[46] da Silva AF, Araujo JMG, de Brito SB, Santos JP, Ferreira AG, Neves PSO (2024). Highly divergent and diverse viral community infecting sylvatic mosquitoes from Northeast Brazil. J Virol.

[47] Aragão CF, Ribeiro LR, Silva SP, Nascimento BLS, Nunes JP, Oliveira TMFS (2023). Shotgun metagenomic sequencing reveals virome composition of mosquitoes from a transition ecosystem of north-northeast Brazil. Genes.

[48] Njabo KY, Cornel AJ, Sehgal RNM, Loiseau C, Buermann W, Harrigan RJ (2009). Coquillettidia (Culicidae, Diptera) mosquitoes are natural vectors of avian malaria in Africa. Malar J.

[49] Diaz-Nieto LM, Macia A, Parisi G, Farina JL, Vidal-Dominguez ME, Perotti MA (2013). Distribution of mosquitoes in the south east of Argentina and first report on the analysis based on 18S rDNA and COI sequences. PLoS One.

[50] Ternovoi VA, Gladysheva AV, Sementsova AO, Zadorozhny AM, Volkova NN, Chausov EV (2023). The viromes of mosquitoes from the natural landscapes of western Siberia. Viruses.

[51] Ripoll L, Iserte J, Cerrudo CS, Presti D, Serrat JH, Poma R (2025). Insect-specific RNA viruses detection in field-caught Aedes aegypti mosquitoes from Argentina using NGS technology. PLoS Negl Trop Dis.

[52] Minkeu FN, Vernick KD (2018). A systematic review of the natural virome of Anopheles mosquitoes. Viruses.

[53] Hernandez-Valencia JC, Muñoz-Laiton P, Gomez GF, Correa MM (2023). A systematic review on the viruses of Anopheles mosquitoes the potential importance for public health. Trop Med Infect Dis.

[54] Moonen JP, Schinkel MP, van der Most T.Miesen P.Vogels CBF.Koenraadt CJM (2023). Composition and global distribution of the mosquito virome - A comprehensive database of insect-specific viruses. One Health.

[55] Pavon JAR, Braun LM, Silva IT, Araujo DB, Hamerly T, Durigon EL (2024). Disclosing the virome of Aedes, Anopheles and Culex female mosquitoes, Alto Pantanal of Mato Grosso, Brazil, 2019. Virology.

[56] Heinisch MR, Diaz LA, Salgado BB, Aviles G, Garcia G, Cusatis D (2023). Fauna and virological investigation of mosquitoes in urban parks in São Paulo, Brazil. J Am Mosq Control Assoc.

[57] Nunes JP, Reis LAM, Freitas MNO, do Nascimento BLS, das Chagas LL, da Costa HHM (2023). First isolation and genome sequence analysis of West Nile virus in mosquitoes in Brazil. Trop Med Infect Dis.

[58] Rutledge CR, Day JF, Lord CC, Stark LM, Tabachnick WJ (2003). West Nile virus infection rates in Culex nigripalpus (Diptera Culicidae) do not reflect transmission rates in Florida. J Med Entomol.

[59] Atoni E, Wang Y, Karungu S, Waruhiu C, Zohaib A, Obanda V (2018). Metagenomic virome analysis of Culex mosquitoes from Kenya and China. Viruses.

