# Supplementary material for: Viral metagenomics in mosquitoes as potential vectors of arboviruses in the Colombian Caribbean: characterisation of a “core” regional RNA virome
Source: Mem Inst Oswaldo Cruz. 2026 Jan 12;120:e250131. doi: 10.1590/0074-02760250131 (PMC12799220; doi:10.1590/0074-02760250131)
Supplement: Supplementary material [file 1678-8060-mioc-120-e250131-s.pdf]

This study characterised the virome of mosquitoes in the Colombian Caribbean, revealing a predominance of insect-specific viruses (ISVs) across all species, which form the core regional viromedefined as viruses consistently present across species and seasons. To elucidate the ecological context of these findings, Supplementary data (Table) integrates viral diversity, literature comparisons, and ecological variables for each mosquito species. *Mansonia titillans* and *Coquillettidia nigricans* exhibited the highest viral richness (38 and 21 species, respectively), with ISVs like *Aedes aegypti* to virus 1 and 2, Picornaviridae, and Rhabdoviridae dominating the core virome. These findings align with studies in Brazil and Argentina, where Flaviviridae and Bunyavirales were reported in *Mansonia* and *Coquillettidia*. Ecological factors, such as riverbank habitats and rainy season conditions (higher temperature and breeding site availability), correlated with increased viral diversity, particularly in *Mansonia* and *Coquillettidia* [Supplementary data (Table)]. No significant arboviruses (*e.g.*, dengue, Zika) were detected, underscoring ISV dominance. The core viromes stability across seasons suggests it modulates vector competence, potentially reducing arbovirus transmission through competitive exclusion. These results highlight the utility of metagenomics in identifying ecological drivers of virome composition and support its application in vector surveillance.

TABLE

Comparison of viral diversity and ecological variables in mosquito species from the Colombian Caribbean

Mosquito species*	Viral families (This study)*	Core virome ISVs	Literature comparison	Habitat type	Season	T (ºC)
Mansonia titillans	Picornaviridae, Flaviviridae, Rhabdoviridae, Orthomyxoviridae, Bunyavirales (38 species)	*Aedes aegypti to virus 1, 2, Astopletus, Cumbaru*	Flaviviridae, Bunyavirales (Brazil)	Riverbanks, vegetation	Rainy	26
Coquillettidia nigricans	Orthomyxoviridae, Parvoviridae, Flaviviridae, Bunyavirales (21 species)	*Aedes aegypti to virus 1, 2, Kaiowa*	Flaviviridae, Rhabdoviridae (Argentina)	Riverbanks, peri-domestic	Rainy	25
Anopheles albimanus	Flaviviridae, Mesoniviridae, Rhabdoviridae	*Aedes aegypti to virus 1, 2*	Mesoniviridae, Peribunyaviridae (Mexico, Colombia)	Residential, vegetation	Rainy	24
Anopheles darlingi	Flaviviridae, unclassified Totiviridae, Iflaviridae	*Aedes aegypti to virus 1, 2*	Totiviridae, Iflaviridae (Brazil, Peru)	Riverbanks	Rainy	25
Culex nigripalpus	Rhabdoviridae, unclassified Riboviria	*Aedes aegypti to virus 1, 2, Nefer*	Rhabdoviridae, Flaviviridae (North America, Brazil)	Peri-domestic	Rainy	26
Culex quinquefasciatus	Flaviviridae, Rhabdoviridae, Metaviridae	*Aedes aegypti to virus 1, 2*	Totiviridae, Baculoviridae (Asia, Africa)	Urban, peri-domestic	Rainy	27

*All viral families are reported according to the International Committee on Taxonomy of Viruses (ICTV). ISVs: insect-specific viruses; T: temperature (°C) refers to the average environmental temperature recorded during the sampling period; scientific names are written in italics following standard taxonomic convention.
